# Construction and analysis of regulatory genetic networks in cervical cancer based on involved microRNAs, target genes, transcription factors and host genes

**DOI:** 10.3892/ol.2014.1814

**Published:** 2014-01-20

**Authors:** NING WANG, ZHIWEN XU, KUNHAO WANG, MINGHUI ZHU, YANG LI

**Affiliations:** 1 Department of Computer Science and Technology, Jilin University, Changchun 130012, P.R. China; 2 Symbol Computation and Knowledge Engineer of Ministry of Education, Jilin University, Changchun 130012, P.R. China

**Keywords:** cervical cancer, transcription factor, microRNA, genetic regulation, network

## Abstract

Over recent years, genes and microRNA (miRNA/miR) have been considered as key biological factors in human carcinogenesis. During cancer development, genes may act as multiple identities, including target genes of miRNA, transcription factors and host genes. The present study concentrated on the regulatory networks consisting of the biological factors involved in cervical cancer in order to investigate their features and affect on this specific pathology. Numerous raw data was collected and organized into purposeful structures, and adaptive procedures were defined for application to the prepared data. The networks were therefore built with the factors as basic components according to their interacting associations. The networks were constructed at three levels of interdependency, including a differentially-expressed network, a related network and a global network. Comparisons and analyses were made at a systematic level rather than from an isolated gene or miRNA. Critical hubs were extracted in the core networks and notable features were discussed, including self-adaption feedback regulation. The present study expounds the pathogenesis from a novel point of view and is proposed to provide inspiration for further investigation and therapy.

## Introduction

It is generally accepted that the accumulation of mutations in critical genes takes major responsibility for the evolution of cancer (1). This abnormal biological phenomenon appears and develops under the control of transcription factors and microRNAs (miRNAs/miRs). In recent years, research on miRNA, small [21–24 nucleotides (nt)] non-coding RNA molecules, has been more widely acknowledged (2). miRNA affects the expression of genes at a post-transcriptional level by binding to complementary sequences on target mRNA, resulting in translational repression or target degradation and gene silencing. miRNA participates in various biological processes, including proliferation, differentiation and apoptosis, independently or together with transcription factors (TFs). Increasing evidence demonstrates that miRNA is of great importance in the development of malignant tumors (3,4). According to this research, miRNAs are involved in numerous significant biological functions and signal transduction pathways, and also in cervical tumorigenesis (5).

A TF is a protein that binds to specific DNA sequences, thereby controlling the flow (or transcription) of genetic information from DNA to mRNA. TFs regulate the expression of genes at a transcriptional level so that they may exert an effect on malignant cell transformation (6,7).

The miRNA and genes, including TFs, involved in cervical cancer are the key elements of the analysis based on computational methods in the present study.

Not all the elements in the complete collection of genes and miRNA can be equally considered in the data mining process. A certain number act abnormally in cervical cancer, which is known as differential expression. Unusual changes similar to this occurring for genes and miRNA may be one of the factors resulting in cancer (8,9).

Genes and miRNA related to cervical cancer were located, but not all of them were differentially-expressed. Considering that the appearance of cancer is ectopic in the human body, the differentially-expressed genes and miRNA can be regarded as the potential factors that have negative carcinogenic effects. Therefore, the present study was focused on the differentially-expressed genes and miRNA, with the assistance of the secondary related ones.

miRNA has multiple connections with various target genes. These genes are indispensable materials in uncovering the role of miRNA in cancers. Numerous resources, including computational predicted methods and experimentally validated databases, provide sufficient data to acquire the associations between miRNA and the corresponding target gene.

Certain miRNAs are located inside genes that are bound to miRNA as their host genes. Rodriguez *et al* indicated that miRNAs are transcribed in parallel with their host transcripts, and two types of transcription (exonic and intronic) were identified that indicate that miRNAs may require slightly different mechanisms of biogenesis (10). Baskerville *et al* indicated that intronic miRNA and its host gene have a closer association than that of exonic miRNA and its corresponding host genes (11). Intronic miRNA and its host genes are usually coordinately expressed in biological progression, and they usually work together to conduct biological functions and affect the alteration of signaling pathways (12). Studies have demonstrated that their differential expression could contribute to the progression of cancer (13,14). Therefore, we suggest that miRNAs can work together with their host gene in the regulatory system.

In the present study, the underlying networks composed of miRNA, target genes, TFs, host genes of miRNA and the regulatory associations represented in human cervical cancer were visualized. Various data was manually collected, including experimentally validated associations between miRNAs and their targets, experimentally validated associations between TFs and miRNAs, and associations of miRNAs and their host genes; they were reserved as fundamental resources to uncover regulatory mechanisms of genes and miRNA in cervical cancer. The differentially-expressed and secondary related genes and miRNAs were collected from databases and the literature. Eventually the networks associated with cervical cancer at three levels were built based on these materials. The first network was the differentially-expressed network constructed from differentially-expressed genes, differentially-expressed miRNA and host genes of differentially-expressed miRNA. The second network was the cervical cancer-related network, which was constructed from related genes, related miRNAs and host genes of cancer-related miRNAs. The third network was the global network, which consisted of all the elements extracted from the basic source data. The differentially-expressed network was the most significant network, and it deserves more attention due to the differentially-expressed characteristics of its constituents. Comparisons were made to find similarities and differences between the three networks, and separate significant regulatory pathways were extracted that play key roles in cervical cancer. The present study revealed certain important core signal networks of TFs, miRNA, targets of miRNA and host genes of miRNA in cervical cancer. The present study will contribute to the understanding of the pathogenesis and the development of therapy for cervical cancer.

## Materials and methods

### Dataset of experimentally validated targeting associations between miRNAs and corresponding target genes

The targeting associations between miRNAs and corresponding genes were acquired based on the data provided by Tarbase 5.0 and miRTarBase (15,16). The list consisted of 6,749 entries describing the targeting interactions of 426 miRNAs and 2,029 genes.

### Dataset of experimentally validated regulating associations between TFs and corresponding miRNAs

The data controlling signal flow from TFs to miRNA was acquired from TransmiR, a manually constructed database of regulating associations between TFs and miRNA (17); this included 862 entries of regulating associations between 153 transcription factors and 220 miRNAs.

### Dataset of miRNAs and host genes

The association mapping host genes and their respective miRNAs were established based on the data from miRBase and the National Centre for Biotechnology Information (NCBI) (http://www.ncbi.nlm.nih.gov/) and miRBase (18). The result contained 1,419 entries between 1,136 host genes and 1,209 miRNAs.

### Differentially-expressed and related miRNAs associated with cervical cancer

The differentially-expressed miRNAs in cervical cancer were mainly extracted from mir2Disease, a manually curated database collecting and reorganizing data on differentially-expressed miRNA in various human diseases (19). Furthermore, supplementary miRNAs were included by a literature search. In the same way, related miRNAs were collected. In total, 11 differentially-expressed miRNAs and 12 related miRNAs were acquired.

### Differentially-expressed genes and related genes associated with cervical cancer

The differentially-expressed genes in cervical cancer were gathered from several sources, including Cancer Genetics Web (http://www.cancerindex.org/geneweb/), the single nucleotide polymorphism database of the NCBI (http://www.ncbi.nlm.nih.gov/snp/) and Kyoto Encyclopedia of Genes and Genomes (20). Again, the literature search was necessary as a complementary resource.

For genes with a secondary correlation to cervical cancer, additional sources were used. The first was the GeneCards database, from which 203 genes of high confidence levels were collected (21). An algorithm called P-match operating was adopted on the concept of pattern matching and weight matrix approaches to identify TF binding sites in DNA sequences (22). From the University of California, Santa Cruz Genome Browser (UCSC), the 1,000-nt promoter region sequences of differentially-expressed genes and target genes of differentially-expressed miRNA were acquired, following an input of P-match (23). The vertebrate matrix and restricted high-quality criterion were chosen, and the products were referred to as part of 1,000-nt TFs in secondary related genes. Again, data mining from the literature search was conducted to provide complementary information. As always, the differentially-expressed genes were regarded as part of the related genes. In total, 38 differentially expressing genes and 241 related genes were collected.

### Construction of networks at three levels

In order to discuss the regulating mechanism between the involved genes and miRNA in the undercover networks of cervical cancer, a series of procedures were designed to investigate the interactions extracted from the data acquired. The raw data became a designed relational model and was used as the basic source for further processing. According to previous processes, the models can be described as follows: *U*_1_ = [(M, *G_T_*)/M targets *G_T_*]; *U*_2_ = [(*G_TF_*, M)/*G_TF_* targets M]; and *U*_3_ = [(*G_H_*, M)/*G_H_* is the host gene of M]. M refers to miRNAs, *G_T_* represents the target gene of corresponding miRNA, *G_TF_* is the transcriptional factor that regulates the expression of miRNA and *G_H_* is miRNA host genes. Respectively, they display the ways in which miRNAs and genes interact with each other. Based on the preinstalled structure, the data was organized into factor pairs marked by interaction types. The search procedure was conducted to extend the original connections to deeper meshwork rather than direct interactions between isolated nodes. The networks are represented at three levels, the differentially-expressed, related and global networks, together with the network with regard to 1,000-nt TFs. Through use of prefabricated programs, the differentially-expressed and related networks could be extracted at corresponding levels with elements filtered as required. The differentially-expressed network lists associations involving differentially-expressed genes and miRNA, together with host genes. Similarly, the related network is of related genes and miRNAs together with host genes.

## Results and Discussion

### Differentially-expressed network in cervical cancer

**Figure 1:**
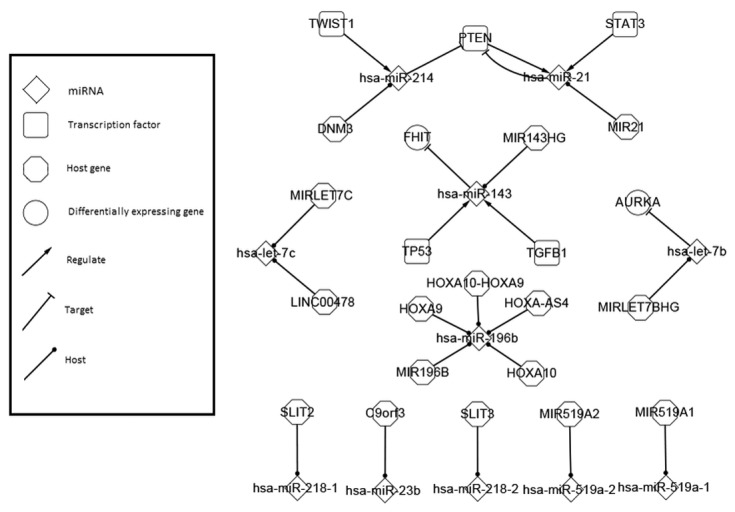
Differentially-expressed network.

The differentially-expressed network is primarily discussed in the present study due to its possession of differentially-expressed biological factors that have been experimentally validated in cervical cancer. The visual representation is shown in Fig. 1.

Seven genes and 10 miRNAs appearing in Fig. 1 were experimentally validated as differentially-expressed in cervical cancer. The sub-network centering on PTEN was the principal component in the network. TWIST1, hsa-miR-214, PTEN and hsa-miR-21 bind together as an ordered control chain. hsa-miR-21 targeted its regulator PTEN, which formed a self-adaption feedback loop, bringing a balance mechanism and involving STAT3 and MIR21 in the system as participants, similar to DNM3. Consequently, two host genes, two transcription factors and one miRNA affected the expression of PTEN and hsa-miR-21. At the same time, these two core factors controlled each other. The circling between hsa-miR-21 and PTEN made them dominant and dominating factors simultaneously, and turned the path to a bidirectional net crux.

According to the organized data, hsa-miR-21 was found to be differentially-expressed in ≥16 carcinomas and PTEN was differentially-expressed in 13. miR-21 is regarded as one of the oncomiRs contributing to tumor cell growth (24,25). With the famous tumor suppressor, PTEN, as a bridge, hsa-miR-214 was involved in the regulatory balance between has-miR-21 and PTEN (26). miR-214 has been demonstrated to inhibit cell growth, migration and invasion (27). These three significant biochemical cancer-related factors are expressed abnormally in a cervical tumor organism and bind together closely as a distinctive sub-system (28–30).

In another partial network centered on hsa-miR-143, TP53 and TGFB1 jointly has been shown to regulate hsa-miR-143, with a resulting signal flow to FHIT. As a generally acknowledged tumor suppressor standing out in various cancer investigations, TP53 may be a starting point for further discussion (31).

### Cervical cancer-related network

**Figure 2:**
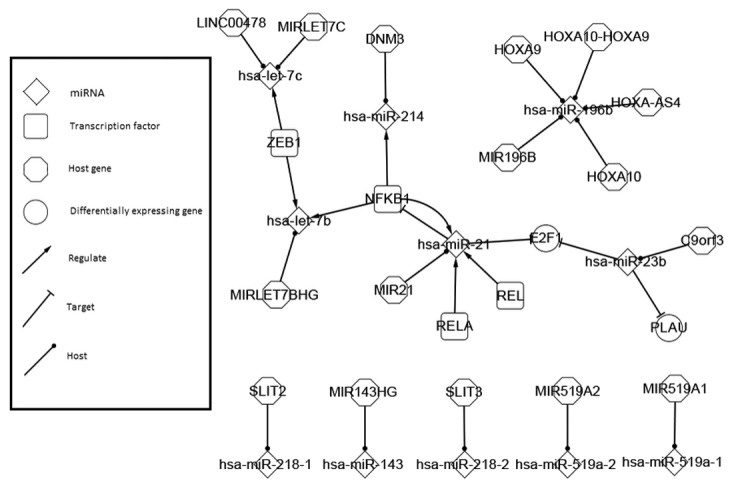
Cervical cancer-related network.

The cervical cancer-related network expanded the differentially-expressed network by absorbing more genes and miRNAs whose association with cervical cancer are not as close as those of the differentially-expressed ones. The network is demonstrated in Fig. 2.

The related network was of a much larger scale and of higher complexity than the differentially-expressed network. Certain factors were prominent, including the miRNAs of hsa-miR-21, hsa-miR-23b, hsa-miR-34, hsa-miR-143, hsa-let-7c and hsa-let-7c, and the TFs of PTEN, TP63, MYC, TP53 and KRAS.

Two more self-adaption feedbacks were identified upon hsa-let-7c, hsa-miR-34 and MYC. MYC acted as the TF of hsa-let-7b and hsa-let-7c, which target KRAS. This is accordant with the outcome that KRAS mutation-positive lung cancer, displaying high levels of MYC, could be treated by inhibiting MYC transactivation function (32). Also, MYC overexpression may disturb the tumor suppressor capability of hsa-let-7c (33). Meanwhile, another tumor suppressor, hsa-miR-34, holds a local balance adjustment system with MYC as well (34).

In a similar way, the related network could be used to identify more possible core factors, sub-networks and motifs.

### Global network of cervical cancer

The global network contained all the factors and associations involved with miRNA-gene targeting associations, TF-miRNA regulating associations and host gene-miRNA hosting associations. The network accommodated the experimentally validated signaling transmissions and potential routes for further investigations.

### Comparison and analysis on features of the 1,000-nt TF network

**Figure 3:**
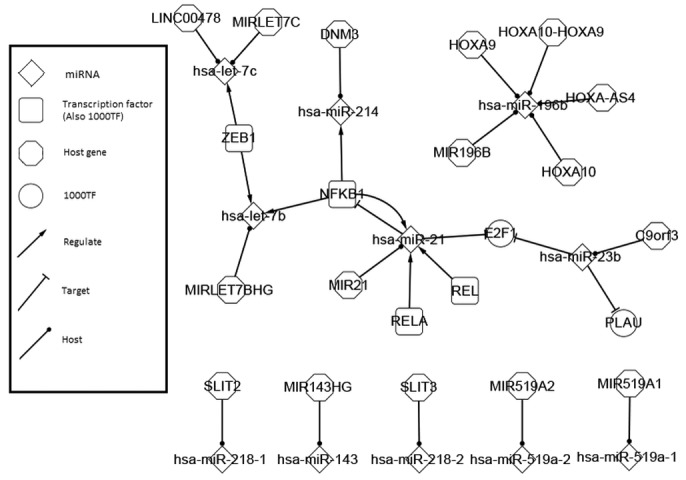
1,000-nucleotide (nt) transcription factor (TF) network.

The interactions around the TFs that were acquired previously and the base sequences of 1,000-nt were evaluated; they were integrated with host genes and differentially-expressed miRNAs to bring 1,000-nt TFs into the network system of cervical cancer for overall clarification. The network is shown in Fig. 3.

As always, self-adaption feedback regulation was assessed first; this existed between NFKB1 and hsa-miR-21 in this circumstance. NFKB1 regulates hsa-miR-21, hsa-miR-214 and hsa-let-7b. As discussed in the differentially-expressed network, hsa-miR-21 and hsa-miR-214 are core biological factors in the cervical cancer network and therefore, the reoccurrence here strengthens the value of these particular miRNAs. With the help of hsa-miR-21 and hsa-miR-214, NFKB1 takes part in the sub-system where PTEN and has-miR-21 act as cores.

The present study defined an organized data system and matched methods that can be computer-applied and transplanted easily for the discussion of other cancer types.

Numerous genes and miRNAs with corresponding specific biological features in cervical cancer were organized into the form of associations representing their interactions with each other as regulators or as those being regulated. The three levels of networks demonstrated direct and indirect associations among transcription factors, miRNAs, target genes and host genes in different degrees of correlation with cervical cancer.

The networks provide core effective systems in cervical cancer, particularly that of hsa-miR-214, PTEN and hsa-miR-21. The methods used in the present study could be utilized in full for further investigations on cancer-related networks.
